# Effect of Locust Bean Gum-Sodium Alginate Coatings Combined with High CO_2_ Modified Atmosphere Packaging on the Quality of Turbot (*Scophthalmus maximus*) during Refrigerated Storage

**DOI:** 10.3390/polym13244376

**Published:** 2021-12-14

**Authors:** Jie Cao, Wenru Liu, Jun Mei, Jing Xie

**Affiliations:** 1 College of Food Science and Technology, Shanghai Ocean University, Shanghai 201306, China; m190300743@st.shou.edu.cn (J.C.); m180300684@st.shou.edu.cn (W.L.); 2 National Experimental Teaching Demonstration Center for Food Science and Engineering, Shanghai Ocean University, Shanghai 201306, China; 3 Shanghai Engineering Research Center of Aquatic Product Processing and Preservation, Shanghai 201306, China; 4 Shanghai Professional Technology Service Platform on Cold Chain Equipment Performance and Energy Saving Evaluation, Shanghai 201306, China

**Keywords:** turbot, active coating, modified atmosphere packaging, total volatile basic nitrogen, shelf life

## Abstract

This research was conducted to investigate the effect of active coatings composed of locust bean gum (LBG) and sodium alginate (SA) containing daphnetin emulsions (DEs) combined with modified atmosphere packaging (MAP) on the microbiological and physicochemical properties of turbot during 4 °C refrigerated storage for 32 days. The results revealed that LBG-SA-DE coatings together with high CO_2_ MAP (60% CO_2_/35% N_2_/5% O_2_) maintained the total viable count (TVC) of H_2_S-producing bacteria in 4–6 lg CFU/g, which is lower than the limit (7 lg CFU/g). In addition, LBG-SA-DE coatings together with high CO_2_ MAP (60% CO_2_/35% N2/5% O_2_) inhibited the production of odor compounds, including thiobarbituric acid (TBA), trimethylamine-nitrogen (TMA-N), K value, and total volatile basic nitrogen (TVB-N). The low-field NMR analysis (LF-NMR) and magnetic resonance imaging (MRI) indicated that LBG-SA-DE coatings together with high CO_2_ MAP (60% CO_2_/35% N_2_/5% O_2_) treatments could delay the release of water located in muscle fiber macromolecules or convert it into free water based on muscle fiber destruction, thus maintaining the water content and migration. The results of the sensory evaluation showed that turbot treated with LBG-SA-DE coatings together with MAP could maintain its freshness during refrigerated storage.

## 1. Introduction

Turbot (*Scophthalmus maximus*) is popular for its extraordinarily palatable flavor, which has prominent economic and nutritional value [1]. Fresh turbot is a highly perishable food due to its high protein content and water activity, and the organoleptic properties of fish deteriorate with storage due to chemical and biological changes [2]. The shelf life of refrigerated fish is limited, mainly owing to the metabolic activities of spoilage microorganisms [3]. Controlling the growth of spoilage bacteria is essential to extending the shelf life of turbot during storage, processing, and distribution. Recently, studies have concentrated on active coatings containing natural preservatives and MAP to extend the shelf life of fish [4,5].

Sodium alginate (SA) is an alginate isolated from brown algae, and it is a polymer composed of L-mannuronic acid and D-glucuronic acid [6,7]. There are a large number of –COONa and –OH reactive groups in the SA molecule; it has excellent biocompatibility and degradation properties for the preparation of SA-based active films [8]. However, due to the strong hydrophilic properties of SA, it is sensitive to humidity, and single SA films are brittle, and weak in strength and elasticity [9]. A common method to improve the performance of SA films is by compounding with other substances such as cellulose and chitosan [10,11]. Locust bean gum (LBG) is an endosperm extract from the seeds of *Sophora japonica*, and is a polysaccharide with galactose and mannose as structural units [12,13]. LBG is colorless and odorless with high viscosity, and good salt resistance, stability, and rheological properties. Due to the good gel synergistic effect of LBG with agar, carrageenan, and other hydrocolloids, it is often used as a thickening agent in combination with other edible gums [14,15].

The addition of natural preservatives to active coatings has attracted increasing attention [16]. Daphnetin is a coumarin derivative extracted from Daphne koreana Nakai, which has been reported to have antimicrobial and antioxidant effects [17,18]. Previous studies have shown that high concentrations of CO_2_ can extend the shelf life of fish products by decreasing the pH in microorganisms and extracting phospholipids and hydrophobic compound fractions from cell membranes, thereby causing inactivation of aerobic bacteria and filamentous fungi [19,20,21]. In this study, active cling films were combined with modified atmosphere packaging the preservative effect of LBG-SA active coatings containing different concentrations of DE in combination with MAP treatment on turbots was evaluated; and the microbiological analysis, TVB-N, K value, TBA, TMA, moisture distribution, and migration were determined.

## 2. Materials and Methods

### 2.1. Additives

Daphnetin (HPLC grade, 90.0%) was purchased from Tokyo Chemical Industries, Ltd. (Tokyo, Japan); sodium alginate and locust bean gum were purchased from Aladdin Biochemical Technology (Shanghai, China). LBG-SA active coatings containing daphnetin emulsions were prepared by Liu et al. [22]. The concentrations of daphnetin emulsions (DEs) used in this study were 0.16, 0.32, and 0.64 g/L; the concentration of MAP packaging in this experiment was 60% CO_2_/35% N_2_/5% O_2_ [23]. These coatings were labeled LBG-SA-0DE, LBG-SA-0.16DE, LBG-SA-0.32DE, and LBG-SA-0.64DE respectively.

### 2.2. Preparation of Turbot Samples

A total of 60 fresh turbot samples (600 ± 50 g, body weight) were purchased from Shanghai Luchao Port Aquatic Products Market (Shanghai, China). Fifty turbots were randomly divided into five groups, as listed in Table 1. Then, the packaged fish were transferred to 4 °C refrigerators for storage. Refrigerated turbots were randomly selected for analysis on the 0th, 4th, 8th, 12th, 16th, 20th, 24th, 28th, and the 32nd day. All experiments were repeated three times.

### 2.3. Microbiological Analysis

An amount of 10 g turbot flesh was mixed with 90 mL of sterilized saline, and 1 mL of the bacterial solution was serially diluted 10 times for microbiological analysis [24,25]: (i) for determination of the total viable count (TVC) on plate count agar medium, the sample was cultivated at 30 °C for 2 days; (ii) for determination of *Pseudomonas* spp. on Pseudomonas CFC selective agar medium, the sample was cultivated at 30 °C for 2 days; (iii) for determination of H_2_S-producing bacteria on iron agar medium, the sample cultivated at 30 °C for 3 days. The logarithm of the average number of colony forming units (CFU) on the solid medium plate with 30–300 colonies was recorded as the final calculation result.

### 2.4. Water Migration

The low-field NMR analysis (LF-NMR) and magnetic resonance imaging (MRI) analyses were performed following the method of Wang et al. [26]. The back flesh of turbot was sampled for measurement. The fish block was imaged by an LF-NMR instrument, and the proton density map was obtained after mapping and pseudo-color analysis.

### 2.5. Analysis of Thiobarbituric Acid (TBA)

An amount of 5 g turbot fish flesh was mixed with 25 mL of 20% trichloroacetic acid and 20 mL of distilled water. After homogenation and standing for 1 h, the supernatant was taken after centrifugation for 10 min at 8000 r/min and the volume was fixed to 50 mL. After shaking well, 5 mL of liquid was mixed with 5 mL of 0.02 mol/L thiobarbituric acid in a water bath at 100 °C for 20 min, then the absorbance was measured at 532 nm with a Multiskan FC enzyme labeler (Thermo Fisher Scientific Co., Shanghai, China); the result is expressed as mg MDA/kg.

### 2.6. Determination of Total Volatile Basic Nitrogen (TVB-N)

The TVB-N value was measured with reference to Valdez’s method [27]: a 25 mL filtrated sample was added to the distillation tube, and steam distillation was performed with a Kjeldahl apparatus (Kjeltec8400, Foss, Hilleroed, Denmark). The results are expressed as mg N/100 g.

### 2.7. Determination of Trimethylamine-Nitrogen (TMA)

The determination of TMA was performed following the method of Yu [28]. The TMA-N value was calculated by the standard curve of TMA (purity > 98%; Sinopharm Chemical Reagent Co., Ltd., Shanghai, China) and expressed as mg N/100 g of turbot sample.

### 2.8. K Value

The ATP-related compounds were measured by HPLC (Waters 2695, Milford, CT, USA) according to Li et al. [4]. The K value was calculated as follows:

(1) $$K\;\mathrm{value}\;(\%)=\frac{\mathrm{HxR}+\mathrm{Hx}}{\mathrm{ATP}+\mathrm{ADP}+\mathrm{AMP}+\mathrm{IMP}+\mathrm{HxR}+\mathrm{Hx}}\times 100\;$$

### 2.9. Sensory Evaluation

The sensory evaluation was carried out with reference to GB/T 18108-2008 and the method of Meral et al. [29]. A score of 10 means the best quality, and the lowest score means poor quality

### 2.10. Statistical Analysis

Data were processed by SPSS 22.0 statistical software; the one-way analysis of variance (ANOVA) procedure (International Business Machines Corporation, Armonk, NY, USA), followed by Duncan’s multiple range tests, was adopted to determine the significant difference (*p* < 0.05) between treatments. The results are expressed as mean ± SD.

## 3. Results and Discussion

### 3.1. Microbiological Results

**Figure 1:**
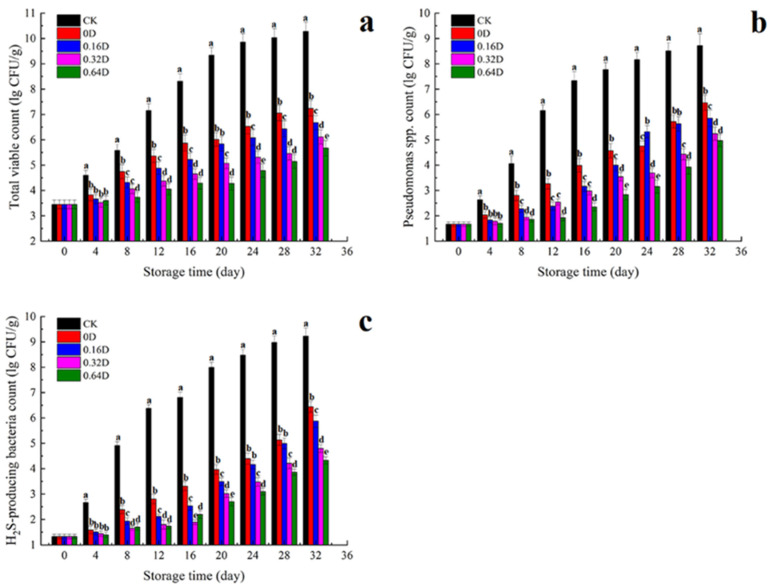
Changes in TVC (**a**), *Pseudomonas* spp. counts (**b**), and H2S-producing bacteria counts (**c**) of turbots during refrigerated storage. Different superscript letters a–e indicate significant differences (*p* < 0.05).

Figure 1 shows the changes in TVC, *Pseudomonas* spp., and H_2_S-producing bacteria in turbots. The TVC counts of all samples increased during refrigerated storage, though the CK samples showed the fastest increase in TVC counts. The TVC count of fresh turbot in this experiment was 3.45 lg CFU/g, which is lower than the initial acceptable amount for fresh fish (4 lg CFU/g), indicating that the fresh turbot samples were of good quality [30]. The TVC count of CK samples reached 7.15 lg CFU/g on the 12th day, exceeding the limit of acceptable microorganisms in marine fish (7 lg CFU/g) [31]. The growth rates of TVC in the other treated samples were lower than those of the CK samples, and the TVC count of LBG-SA-DE treated samples did not exceed the upper limit after 32 days of refrigeration storage. The growth trends of *Pseudomonas* spp. and H_2_S-producing bacteria were similar to that of the TVC counts (Figure 1b,c). At the end of refrigerated storage, *Pseudomonas* spp. and H_2_S-producing bacteria counts in turbots at 0.16D, 0.32D, and 0.64D of treatment remained within the range of 4–6 lg CFU/g and did not exceed the limits. This is due to the significant antibacterial effect of daphnetin. LBG-SA-DE coatings can keep turbot bacterial counts below 7 lg CFU/g at 18 days [23]. Rodrigues et al. suggested that higher CO_2_ and lower O_2_ concentrations in MAP can inhibit microbial growth, thereby reducing spoilage [32]. Kimbuathong et al. also indicated that an increase in CO_2_ (20% to 80%) and a decrease in O_2_ (15% to 5%) in the package effectively delayed microbial growth and inhibited melanosis in Pacific white shrimp [33]. Therefore, LBG-SA-DE coatings combined with high-concentration CO_2_ MAP could have a good inhibitory effect on specific spoilage bacteria.

### 3.2. Water Distribution

**Figure 2:**
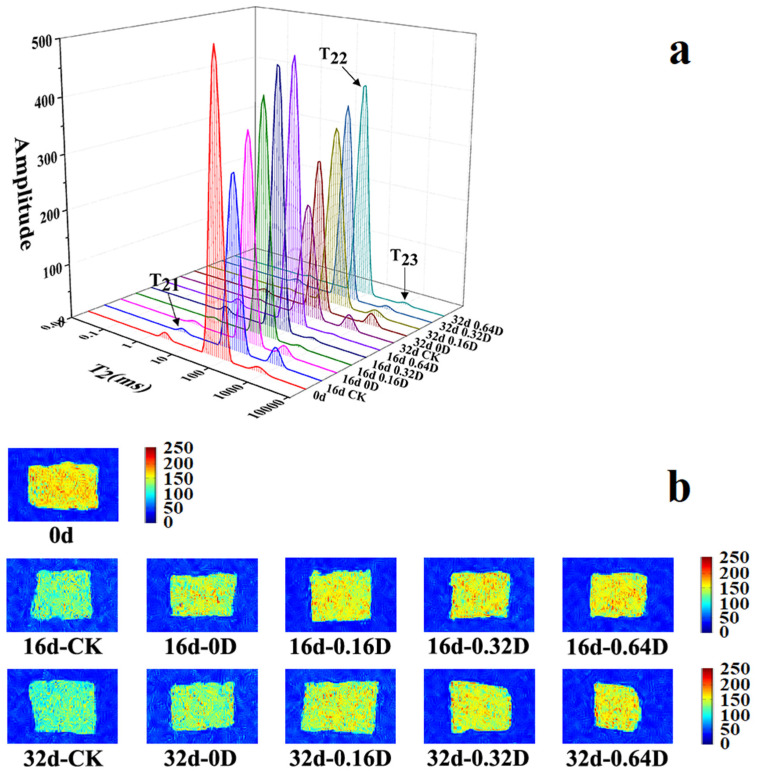
Changes in the water distribution (**a**) and MRI (**b**) of turbots during refrigerated storage.

LF-NMR determined the relaxation time T2 to distinguish the distribution of bound water, free water, and immobilized water in fish flesh [34]. The T2 value reflects the degree of freedom of each moisture, which refers to the degree of combination of each moisture and fish. The smaller the T2 value, the lower the degree of freedom of moisture and the closer the degree of combination. Three peaks appeared in the relaxation pattern of the turbot samples. The three peaks in the spectrum represented the binding water closely related to the protein macromolecule: the peak < 10 ms (T_21_) represents bound water, the peak at 20 to 400 ms (T_22_) represents the immobilized water; the peak >1000 ms (T_23_) represents the free water [35]. In this study, there was no significant difference in T_21_ between turbots; however, T_22_ gradually decreased and T_23_ increased during storage (Figure 2a). The results showed that the changes in free water were more pronounced during refrigerated storage. T_21_ was unaffected by treatment method and storage time due to the fact that water is in the tertiary and stable quaternary protein structure within the highly organized muscle fiber structure [36,37]. CK samples had the lowest immobilized water content. Several studies have shown that water located within the myofibril macromolecules can be released or converted to free water as the myofibrils are destroyed during refrigerated storage [38,39]. Li et al. suggested that higher CO_2_ and lower O_2_ concentrations could reduce the diffusion rate of fixed water to free water in muscle fiber [24]. In addition, this process of water migration was well-reflected in the phenomenon that the LBG-SA-DE coating treatment retarded the rate of change of T_22_ and T_23_ [23]. Above all, LBG-SA-DE active coatings combining with high-concentration CO_2_ MAP effectively delayed the release of water located in muscle fiber macromolecules or converted it into free water based on muscle fiber destruction, maintaining the excellent quality of turbot.

MRI can be used to observe the space and distribution of water in fish flesh [40]. In Figure 2b, red represents high proton density and blue represents low proton density in the pseudo-color images. The color of CK samples on 16th and 32nd days and 0D samples on the 32nd day were bluer and darker, demonstrating moisture of the sample was severely reduced in the CK samples [34]. In addition, compared with the CK and LBG-SA samples, the turbot treated with LBG-SA-DE coatings had lighter brightness, and there was no significant difference between 0.16D, 0.32D, and 0.64D samples. The result showed that LBG-SA-DE coatings combined with high concentration CO_2_ MAP treatment could slow the water loss of turbot during refrigerated storage.

### 3.3. TBA Analysis

**Figure 3:**
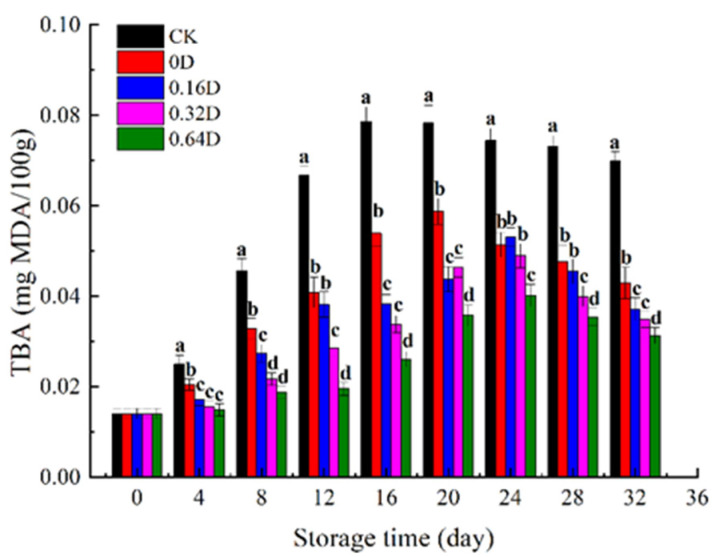
Changes in the TBA of turbots during refrigerated storage. Different superscript letters a–d indicate significant differences (*p* < 0.05).

TBA is an important indicator for determining the oxidation degree of fish fat, and is widely used to measure the degree of oxidation and rancidity of fish fat. When the degree of oxidation is high, the TBA value increases [41]. Figure 3 reveals the variation in TBA during refrigerated storage. The TBA value of fresh turbot was 0.015 mg MDA/kg and increased with storage time. The increase in TBA values is mainly related to the oxidation of fatty acids in fish. During storage, the peroxide decomposition of fish flesh produces volatile substances such as malondialdehyde; the oxidation process is influenced by the O_2_ concentration [42,43]. The decrease in TBA values in the final phase is mainly owing to the interaction of malondialdehyde with other volatile compounds [44]. However, as the refrigeration time increased, the samples treated with LBG-SA-DE coatings combined with high-concentration CO_2_ MAP revealed lower TBA values. The low concentrations of O_2_ inside the packaging inhibited the auto-oxidation of lipid, and the higher CO_2_ concentration inhibited enzymatic hydrolysis. Therefore, the active coatings containing daphnetin emulsions combined with high-concentration CO_2_ MAP could effectively slow the lipid oxidation of turbot.

### 3.4. Changes in TVB-N

**Figure 4:**
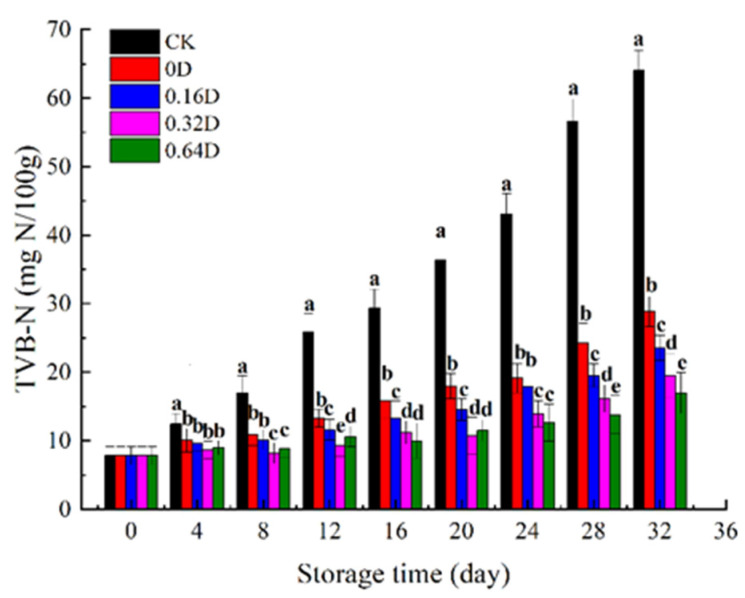
Changes in the TVB-N of turbots during refrigerated storage. Different superscript letters a–e indicate significant differences (*p* < 0.05). Different superscript letters a–d indicate significant differences (*p* < 0.05).

During refrigerated storage, turbot flesh is susceptible to endogenous proteases and microorganisms. Flesh proteins decompose into trimethylamine, amines, and other volatile alkali substances, making their TVB-N values rise. Therefore, the TVB-N value is an important indicator for evaluating fish freshness [45]. Figure 4 reveals changes in the TVB-N values of turbots. The initial TVB-N value of turbot was only 7.81 mg/100 g, indicating the good quality of fresh turbot. Then, the TVB-N values presented an increasing trend, and increased more rapidly in the final phase of storage due to the increase in bacterial activity. The rate of increase in TVB-N values in the CK samples was significantly higher than that of the treated samples (*p* < 0.05). The CK samples showed higher TVB-N values than the LBG-SA-DE treated samples. The TVB-N values in the CK group exceeded the limit value of 25 mg N/100 g at 12th day [46]. While the turbots treated with LBG-SA-DE active coatings still maintained low TVB-N values, the higher the content of DEs in the coatings, the lower the TVB-N value. The lower TVB-N values of turbot samples treated with LBG-SA-DE may be related to the antibacterial effect of daphnetin [35]. Compared to the CK treatment, MAP reduced the oxygen concentration, inhibited bacterial growth, and slowed down spoilage, thus reducing the production of basic nitrogen compounds [47], which is the same trend in variation as shown by the TVC results.

### 3.5. Changes in TMA-N

**Figure 5:**
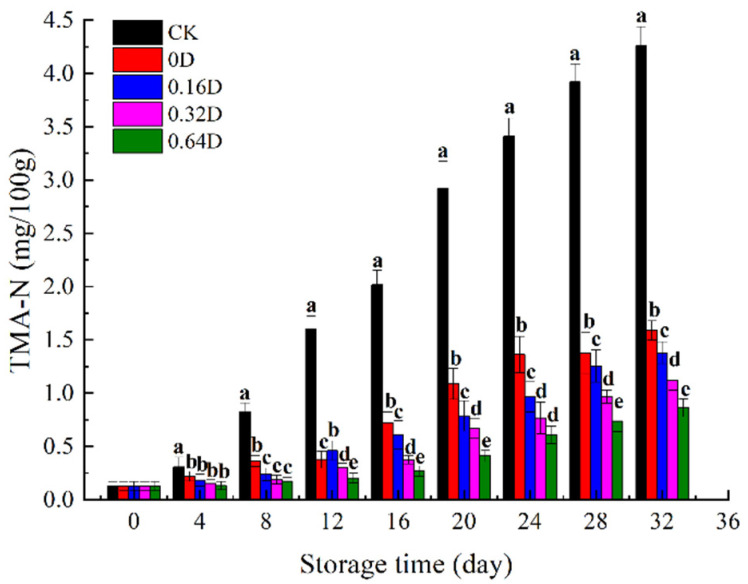
Changes in the TMA value of turbots during refrigerated storage. Different superscript letters a–e indicate significant differences (*p* < 0.05).

During the refrigerated storage of marine fish, specific spoilage bacteria degrade trimethylamine oxides to produce TMA-N, which is the main reason for the fishy off-odor of marine fish [48]. The results showed that the formation of TMA-N occurred in all turbot samples (Figure 5), and turbot samples treated with LBG-SA-DE active coatings combined with MAP had lower TMA-N values. The TMA-N value of fresh turbot was 0.12 mg/100 g. On the 16th day, the TMA-N level of CK samples reached 2.02 mg/100 g, and the samples showed an unpleasant odor due to the high level of TMA-N. Referring to the results of TVC measurement, 1.5 mg TMA-N per 100 g of turbot produced in the CK sample was set as the standard for the onset of spoilage [23]. Referring to this standard, the 0D samples began to deteriorate on the 28th day, while the 0.16D, 0.32D, and 0.64D samples met the edible criteria until the end of the refrigerated storage. On the 32nd day, LBG-SA-DE treated samples presented lower TMA-N values compared to CK samples (*p* < 0.05), which could be attributed to the antibacterial effect of daphnetin on spoilage bacteria, as bacteria are also the main cause of TMA-N formation [49].

### 3.6. K Value

**Figure 6:**
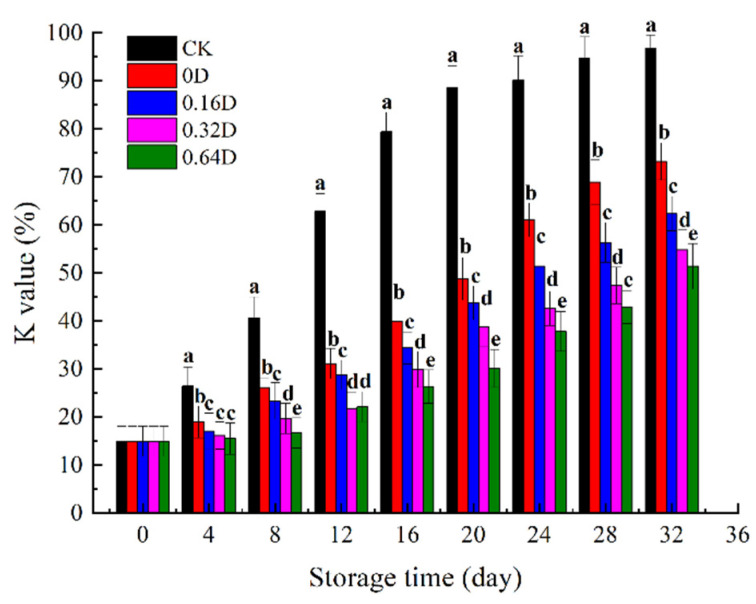
Changes in the K value of turbots during refrigerated storage. Different superscript letters a–e indicate significant differences (*p* < 0.05).

The K value obtained from ATP degradation is an important indicator for evaluating the freshness of fish; 60% is usually considered as the rejection limit of K value [50]. As shown in Figure 6, the K value of fresh turbot was 14.56% and increased in all samples during storage. Similar to the trends in TMA-N and TVB-N, the turbot samples treated with LBG-SA-DE active coating combined with MAP showed a delay in the increase in K values. The K value of the CK sample exceeded 60% on the 12th day, indicating that the turbot had spoiled and deteriorated. Until the 32nd day of refrigeration storage, K values increased to 96.83%, 73.19%, 62.32%, 54.79%, and 51.33% for CK 0D, 0.16D, 0.32D, and 0.64D samples, respectively. The results showed that LBG-SA-DE coatings combined with MAP resulted in the effective inhibition of ATP degradation.

### 3.7. Sensory Evaluation

**Figure 7:**
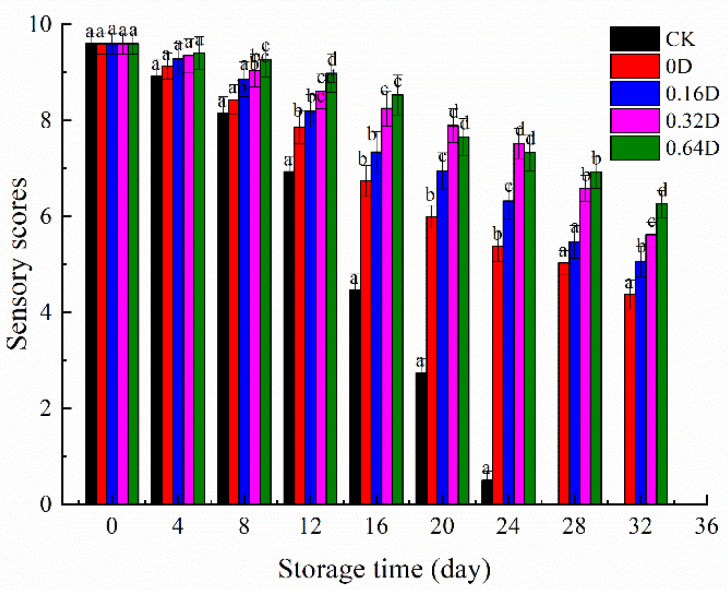
Changes in the sensory scores of turbot samples during refrigerated storage. Different superscript letters a–d indicate significant differences (*p* < 0.05).

As shown in Figure 7, the sensory scores of each group of samples gradually decreased with the extension of storage time, indicating a corresponding decrease in turbot quality. The results of sensory evaluation showed that fresh turbot received the highest ratings for each sensory characteristic at the beginning of the refrigerated storage. The sensory scores of turbot samples decreased, and the LBG-SA-DE-treated samples showed higher scores than CK samples (*p* < 0.05). On the 16th day, the CK score was below the limit of five, which is considered unacceptable for turbot. However, 0.16D, 0.32D, and 0.64D samples exceeded the limitation on the 32nd day, and they remained in good condition during refrigerated storage, presenting similar color and odor to fresh fish and almost no mucus production, with firm and elastic flesh tissue. Therefore, the sensory evaluation showed that LBG-SA-DE coatings combined with MAP could better maintain the quality of turbot during refrigerated storage.

## 4. Conclusions

This study investigated the effect of turbot treated with LBG-SA active coating containing DE (0.16, 0.32 and 0.64 mg/mL, respectively) combined with MAP (60% CO_2_/35% N_2_/5% O_2_) on water migration, fat oxidation, and quality changes during refrigerated storage. The results showed that the number of microorganisms, and TMA-N, TBA, and K values increased in all groups as the storage time increased, but the preservation effect of LBG-SA-DE combined with MAP treatment groups was better than that of the CK group. The high concentration of CO_2_ and daphnetin in the treatment group inhibited the growth of spoilage bacteria and reduce the rate of fat oxidation in fish. According to LF-NMR structural analysis, LBG-SA-DE active coating combined with MAP effectively retarded the degradation of muscle fiber structure. We found that 0.32 or 0.64 mg/mL DE treatment had similar effects in refrigerated storage. Therefore, considering economic and additives, 0.32 mg/mL daphnetin emulsions are recommended to add to maintain the quality of turbot.

## Figures and Tables

**Table 1:** Sample preparation with different daphnetin contents and modes of packaging.

Sample	Daphnetin Content (g/L)	Modes of Packaging
CK	-	Air
0D	-	Modified atmosphere
0.16D	0.16	Modified atmosphere
0.32D	0.32	Modified atmosphere
0.64D	0.64	Modified atmosphere
